# Intestinal Ultrasound for Monitoring Postoperative Crohn’s Disease: A Review and Visual Atlas

**DOI:** 10.1093/ibd/izaf248

**Published:** 2025-12-03

**Authors:** Phillip Gu, Christian Karime, Phillip Fleshner, Katherine Falloon, Taha Qazi, Kenneth Ernest-Suarez, Baldeep Pabla, Joëlle St-Pierre, Gil Y Melmed, Oriana M Damas, Hien Q Huynh, Cathy Lu, Amelia Kellar, the Intestinal Ultrasound Group of the United States and Canada (iUSCAN), Phillip Gu, Christian Karime, Phillip Fleshner, Katherine Falloon, Taha Qazi, Kenneth Ernest-Suarez, Baldeep Pabla, Joëlle St-Pierre, Gil Y Melmed, Oriana M Damas, Hien Q Huynh, Cathy Lu, Amelia Kellar

**Affiliations:** F. Widjaja Inflammatory Bowel Disease Institute, Cedars Sinai Medical Center, Los Angeles, CA, United States; Karsh Division of Gastroenterology and Hepatology, Department of Medicine, Cedars Sinai Medical Center, Los Angeles, CA, United States; Division of Colorectal Surgery, Cedars-Sinai Medical Center, Los Angeles, CA, United States; Division of Gastroenterology, Hepatology, and Nutrition, Digestive Disease and Surgery Institute, Cleveland Clinic Foundation, Cleveland, OH, United States; Division of Gastroenterology, Hepatology, and Nutrition, Digestive Disease and Surgery Institute, Cleveland Clinic Foundation, Cleveland, OH, United States; Department of Medicine, Faculty of Medicine, University of Costa Rica, San Jose, Costa Rica; Division of Gastroenterology and Hepatology, Department of Medicine, Vanderbilt University, Nashville, TN, United States; Division of Gastroenterology and Hepatology, Department of Medicine, University of Calgary, Calgary, Alberta, Canada; F. Widjaja Inflammatory Bowel Disease Institute, Cedars Sinai Medical Center, Los Angeles, CA, United States; Division of Gastroenterology and Hepatology, Department of Medicine, University of Miami, Miami, FL, United States; Division of Pediatric Gastroenterology and Nutrition, Department of Pediatrics, University of Alberta, Edmonton, Alberta, Canada; Division of Gastroenterology and Hepatology, Department of Medicine, University of Calgary, Calgary, Alberta, Canada; Inflammatory Bowel Disease Center, University of Chicago Medicine, Chicago, IL, United States

**Keywords:** intestinal ultrasound, Crohn’s disease, postoperative recurrence, anastomosis

## Abstract

Despite advances in therapeutic strategies, postoperative recurrence (POR) of Crohn’s disease (CD) remains common, underscoring the importance of vigilant and accurate surveillance. Colonoscopy is the gold standard to assess for POR, but it is invasive and can be poorly tolerated by patients. Intestinal ultrasound (IUS) has emerged as a reliable, noninvasive modality for monitoring CD at the point of care and has excellent accuracy for evaluation of POR. However, visualization of the ileocolic anastomosis with IUS can be challenging. This review provides practical guidance for identifying the ileocolic anastomosis and its key sonographic landmarks. It also outlines techniques for assessing the anastomosis with grayscale IUS and discusses strategies for integrating IUS into routine postoperative surveillance of CD.

## Introduction

### Postoperative Recurrence of Crohn’s Disease: A Persistent Challenge in Inflammatory Bowel Disease

Crohn’s disease (CD) is a chronic, relapsing, immunologically mediated inflammatory disorder of the gastrointestinal tract. CD can cause transmural inflammation of gastrointestinal tract from the mouth to anus, resulting in structural bowel damage and complications including inflammatory and fibrostenotic strictures, inflammatory masses, enteric fistulae and/or abscesses. While the increasing use of advanced therapies such as tumor necrosis factor α inhibitors have improved long-term outcomes in CD, 26% of patients will require surgery within 10 years of diagnosis for medically refractory disease and/or CD-related complications.^1^ Despite advanced therapies, ­postoperative recurrence (POR) is common, with up to 55% of patients developing endoscopic evidence of POR within 3 years of surgery and 60% of patients developing recurrent CD-related symptoms within 3 to 4 years.^2^^,^^3^ Ultimately, data show that 30% of CD patients will require a second surgery within 10 years.^1^

Prevention of POR is a key priority in the management of IBD, as repeated surgeries can increase the risk of complications such as adhesions, malabsorption, and short bowel syndrome, which can substantially impair a patient’s quality of life.^4^ As early initiation of CD-directed therapy has been shown to reduce rates of POR, diligent and accurate monitoring for POR is integral to postoperative CD management. Current societal guidelines recommend ileocolonoscopy 6 to 12 months after surgery to assess for POR, and patients should alter or continue current therapy based on endoscopic findings.^5–7^ Although ileocolonoscopy is the gold-standard, it is a costly and invasive procedure that requires considerable coordination and time investment, with need for both bowel preparation and sedation. Moreover, ileocolonoscopy carries procedural risks of complications.^8^ As such, less invasive modalities for monitoring of POR have been proposed, each carrying their own advantages and disadvantages (Table 1). In postoperative CD, cross-sectional imaging such as magnetic resonance enterography (MRE) and computer tomography enterography (CTE) are highly sensitive for detecting POR (90%-93%) with acceptable specificity (67%-78%).^9^ However, MRE and CTE have important limitations, such as high operational costs, limited availability, and need for oral contrast. In addition, radiation exposure is a concern for CTE, limiting its utility in pediatric and pregnant patients. The ability to remain still is also challenging, at times requiring general anesthesia for pediatric patients. Capsule endoscopy offers an alternative modality for assessment of POR with high reported sensitivity, although specificity is limited. Nevertheless, capsule retention and the need for fasting with bowel preparation limits its utility.^10^ Last, fecal calprotectin (FCP) can detect POR but requires patients to collect, store, and deliver stool, leading to a poor completion rate.^11^ As such, an accurate and noninvasive tool for monitoring postoperative CD is a persistent clinical need in the management of IBD.

**Table 1.** Limitations of existing noninvasive monitoring modalities for postoperative CD.

Procedure	Required patient preparation	Limitations
MR enterography	Fasting	Contrast-related allergies Claustrophobia-related distress General anesthesia may be required in pediatrics, which precludes oral contrast Cost Limited availability in some centers
CT enterography	Fasting	Radiation exposure Contrast-related allergies Claustrophobia-related distress
Video capsule endoscopy	Fasting Bowel cleansing	Capsule retention
Fecal calprotectin	Stool collection and storage	Poor completion rate
Intestinal ultrasound	None	Lower sensitivity for detecting mid-small bowel disease Image quality can be impacted by abdominal/body habitus

Abbreviations: CD, Crohn’s disease; CT, computed tomography; MR, magnetic resonance.

### Emerging role of intestinal ultrasound for monitoring postoperative CD

Intestinal ultrasound (IUS) is increasingly utilized as a reliable, noninvasive modality for the accurate monitoring of CD.^12^ IUS can be performed at the point of care and does not require fasting or bowel preparation, making it highly acceptable to patients and easily repeatable.^13^ Moreover, several studies have demonstrated that IUS is an accurate and reliable tool for detecting and monitoring POR, with reported pooled sensitivity and specificity of 97.7% and 83.3%, respectively.^14–16^ Additionally, IUS had comparable accuracy for detecting POR to capsule endoscopy and magnetic resonance imaging.^10^ These results highlight its potential as an effective initial tool for evaluating and surveilling POR.

Among the individual IUS parameters used to assess postoperative disease activity and recurrence in CD, bowel wall thickness (BWT) and presence of mesenteric lymph nodes have the most significant diagnostic yield.^12^^,^^14^^,^^17^ BWT >3mm is an independent predictor of endoscopic POR, and a BWT cutoff ≥5.5 mm has an 84% sensitivity and 98% specificity for detecting severe endoscopic POR (Rutgeerts ≥i3).^14^^,^^16^ Increased bowel wall color Doppler signal (CDS), which reflects hyperemia, is often present in patients with POR but is not an independent predictor for POR.^14^ Other IUS parameters, such as inflammatory fat and disruption of bowel wall echo stratification, are less sensitive for POR but are more specific for identifying severe endoscopic POR (Rutgeerts ≥i3).^18^^,^^19^ Of note, increased BWT in postoperative CD may be secondary to fibrosis or chronic postoperative changes rather than to inflammation, so the presence of CDS can help distinguish between active vs chronic inflammatory changes.^20^ With regard to elastography, there are limited data regarding its utility and ability to differentiate active inflammation from fibrosis.^21^ Similarly, mesenteric lymphadenopathy can be present in other gastrointestinal disorders, thus making it a nonspecific parameter for detecting POR.^22^ For these reasons, utilizing several IUS parameters in combination (such as BWT, mesenteric lymphadenopathy, and CDS) as well as including IUS as an adjunct with other noninvasive modalities such as FCP increases the diagnostic yield.^14^

Although studies have demonstrated the clinical utility of IUS in detecting and monitoring POR in CD, visualization of the ileocolic anastomosis remains more challenging compared with other intestinal segments. This review aims to provide practical guidance for identifying the ileocolic anastomosis and its key sonographic landmarks. We also describe our techniques for assessing the anastomotic bowel with grayscale IUS. Additionally, we describe the role of IUS integration into postoperative surveillance of CD.

## Classification of Ileocolic Anastomosis

This section outlines the predominant surgical techniques employed for anastomosis following ileocolic resection (ICR) in CD, including end-to-end anastomosis (ETEA), side-to-side anastomosis (STSA), end-to-side anastomosis (ETSA), and Kono-S anastomosis (KSA) configurations.

### End-to-end anastomosis

ETEA involves connecting the distal end of the ileum to the proximal end of the colon as a continuous tube. To achieve continuity in the setting of the size discrepancy between the small and large bowel, the small bowel is either enlarged with an antimesenteric incision so that its diameter aligns with the large bowel lumen or by tailored resection of a part of the staple line of the cross-stapled colon.^23^ As a result of this mucosa-to-mucosa approximation, the ETEA anastomosis heals by primary intention.^23^

### Side-to-side anastomosis

**Figure 1.**
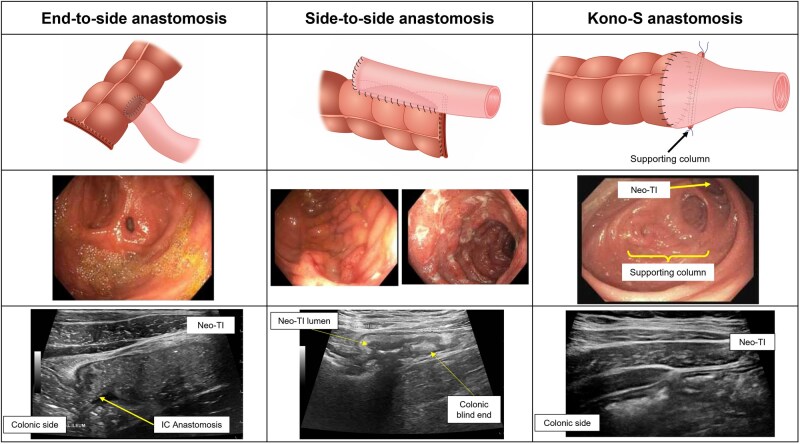
Ileocolic anastomosis configurations with corresponding appearance on endoscopy and intestinal ultrasound. IC, Ileocolic anastomosis; Neo-TI, neoterminal ileum.

**Figure 2.**
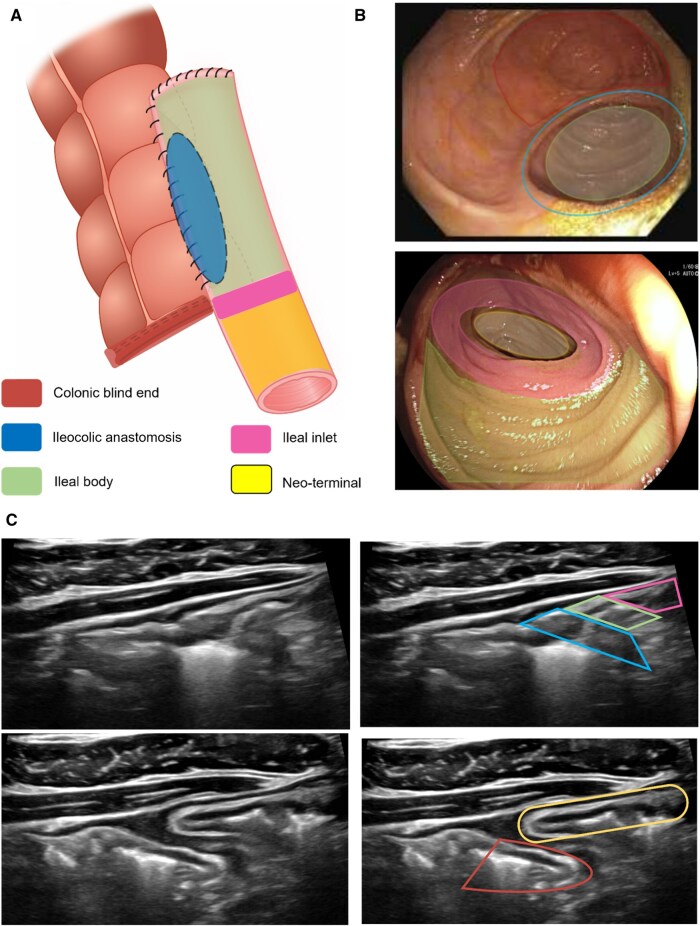
Ileocolic anastomosis landmarks. (A) The anatomic landmarks are highlighted with their corresponding colors on (B) ileocolonoscopy and (C) intestinal ultrasound: colonic blind end (red), ileocolic anastomosis (blue), ileal body (green) ileal inlet (pink), and neoterminal ileum (yellow).

An STSA (Figure 1) is constructed by approximating the ileum and colon side by side, usually along the antimesenteric border, and connecting them using a stapled technique. An STSA can be configured in 1 of 2 orientations: antiperistaltic or isoperistaltic. In the antiperistaltic STSA, one of the intestinal segments is oriented in the opposite direction of its normal peristaltic flow. This creates a segment in which the flow of contents is reversed, with the intent being to create a pseudo-valve effect to help regulate fecal flow. In the isoperistaltic STSA, the intestinal segments are oriented so that the direction of peristalsis and fecal flow remains the same in the joined segment.^24^ The isoperistaltic STSA is similar to the antiperistaltic STSA in terms of convenience and complexity but requires less intestinal mobilization to overlap and anastomose.^25^  Figure 2 illustrates examples of the appearance of an antiperistaltic STSA on endoscopy and IUS. Supplementary Media 1 and 2 provide additional videos of STSA on IUS.

### End-to-side anastomosis

The ETSA (Figure 1) is useful in cases when joining 2 portions of the intestine with different luminal diameters and can be performed either as a handsewn or stapled anastomosis. In this configuration, the end of the ileum is attached to the side of the colon. Supplementary Media 3 and 4 provide additional videos of STSA on IUS.

### Kono-S anastomosis

The KSA (Figure 1) is a recently introduced configuration that uses a “support column” below a side-to-side antimesenteric anastomosis in order to maximize blood supply and exclude mesentery from the anastomotic area in an effort to prevent disease recurrence at the anastomotic site.^26^ After resection of the inflamed segment of ileum, the KSA is constructed by transversely closing the cut ends of the ileum and colon to create blind stumps and constructing a supporting column. Longitudinal incisions are then made along the antimesenteric side of the ileum and colon and the enterotomies are stapled or sutured closed. By excluding the mesentery at the anastomosis, the KSA was suggested to reduce POR, but studies have reported mixed results.^27–29^

## Anastomotic Landmarks on IUS

The original Rutgeerts score was developed to predict risk of symptomatic POR based on lesions observed in patients with ETEA, which only contained the ileocolic anastomosis and neoterminal ileum.^3^^,^^30^ Subsequent advances in surgical techniques with STSA and ETSA have created new anatomic locations such as the colonic blind loop, ileal body and ileal inlet. Hence, depending on the anastomoses, ileocolic anastomosis (ICA) landmarks on IUS will vary significantly. Because STSA and ETSA are the most common anastomoses in modern practice, we have outlined landmarks on IUS based on these configurations. Figure 2 illustrates the landmarks of an isoperistaltic STSA on endoscopy and IUS. An ETSA has similar landmarks except for the colonic blind loop. Key landmarks to measure on IUS when monitoring postoperative CD are at the anastomosis and neoterminal ileum. Current evidence does not differentiate prognostic significance of lesions in the ileal body, ileal inlet, or neoterminal ileum proper.^31^ Thus, to be pragmatic, we recommend measurements for the neoterminal ileum starting 1 cm proximal to the anastomosis as well as the anastomosis itself.

## IUS Technique in Postoperative CD

This section focuses on the IUS technique specifically in postoperative CD; the general IUS technique^32^ is beyond the scope of this review. In the postsurgical abdomen, the ICA cannot be identified with traditional landmarks that are used to visualize a surgically naïve terminal ileum (ie, cranial to the right iliac vessels and psoas muscle). For these reasons, starting with a lower-frequency convex array probe (3-5 MHz), which provides visualization at a greater depth but with lower resolution, will aid in identifying and tracing the colonic segments proximally and localizing the ICA efficiently and accurately. Identifying the exact location of the anastomosis may not always be possible depending on the patient’s anatomy and orientation of the bowel segment in the abdomen, but its approximate location can be localized by identifying the region where the colon is directly adjacent to small bowel in the abdomen. Following the bowel contents (often represented by a line of bowel gas) entering the distal colon from the neoterminal adds further confidence for identifying the anastomosis (Supplemental Figure 1). Once the ICA is localized, the convex and/or the higher-frequency linear array probe (5-15 MHz) may be used to perform the formal exam and obtain measurements. While the linear array probe provides greater resolution, it may have difficulty visualizing anastomoses that are located >4 cm deep to the abdominal wall from the skin, so convex array probes may be better in these patients. For the ICA, conventional IUS parameters (ie, BWT, CDS, bowel stratification, and inflammatory fat) should be assessed at the ICA itself, proximal and distal bowel segments (including the neoterminal ileum located at least 1 cm proximal to the anastomosis) as well as the colonic blind side and/or the ileal blind side if present. In situations in which the provider needs to differentiate between inflammatory vs chronic changes of strictures, CDS can be helpful with CDS modified Limberg score (mLS) >2, suggesting a significant inflammatory component. Given the absence of data supporting different ICA configurations require different measurements relative to the ICA proper, we recommend following the same technique described previously.

There are limited but encouraging data that small intestinal contrast ultrasound (SICUS) using oral contrast can improve the sensitivity of detecting POR.^16^ In a prospective study of 40 subjects, the sensitivity and specificity for detecting POR was 77% and 94% with traditional IUS vs 82% and 94%, respectively, with SICUS.^33^ SICUS improves bowel distention and promotes motility for better assessment of the bowel wall and detection of known/suspected CD complications such as strictures and fistulas. However, it continues to have limited use in practice. For postoperative CD, SICUS is particularly helpful in patients with known or suspected ICA stenosis. There are various protocols used with varying volumes of polyethylene glycol, ranging typically from 250 to 500 cm^3^ between 30 to 60 minutes before IUS examination. Supplementary Media 5 provides an example of SICUS with bowel effluent traversing the ileocolic anastomosis into the colon.

Detecting enteroenteric (Supplementary Media 6) and colocolonic anastomosis (Supplementary Media 7) can be achieved with similar strategies as the ICA but requires focused tracing of the bowel to detect intramural features that help localize the anastomosis. First, detecting the staple line (Supplementary Figure 2) can support localization of the anastomosis. Second, identifying localized defects that disrupt the typical smooth linear pattern of the bowel wall can also help. Finally, the anastomosis may be reflected by a very short bowel segment with prominent muscularis propria layer. When evaluating these anastomoses for POR of CD, the same location and types of measurements as the IC anastomosis apply.

### Parameters for activity measurement

There is currently no validated IUS scoring index for evaluation of postoperative CD activity. Available scores combine several IUS parameters with score-specific weighting to achieve greater accuracy (Supplementary Table 1). Three segmental IUS scores have been developed and internally validated with the Simple Endoscopic Score for Crohn’s Disease (SES-CD) in adults and include BWT and CDS: the Simple Ultrasound Score for Crohn’s Disease (SUS-CD),^34^ Simple Ultrasound Activity Index for Crohn’s Disease (SUAS-CD),^35^ and Bowel Ultrasound Score (BUSS).^36^ A fourth score, the Simple Pediatric Activity Ultrasound Score, includes inflammatory fat, BWT, and CDS.^37^ Although developed specifically for the pediatric CD population, it lacks objective validation against SES-CD. Last, the International Bowel Ultrasound Segment Activity Score (IBUS-SAS) combines the parameters BWT, CDS, inflammatory fat, and bowel wall stratification (BWS).^38^ The latter was developed based on international expert consensus of inflammatory activity parameters.

In a recent large cross-sectional study, accuracy of segmental IUS parameters and the IUS indices (IBUS-SAS, BUSS, SUS-CD, SUAS-CD, and Simple Pediatric Activity Ultrasound Score) were compared with endoscopic SES-CD in young adults with CD without prior bowel surgery. In terms of detecting segmental endoscopic remission (defined as SES-CD ≤2) as well as moderate-to-severe endoscopic inflammation (defined as SES-CD ≥6), BWT, BWs, and IUS scores all demonstrated a similarly high degree of accuracy.^39^ More specifically, BWT had similar area under the receiver-operating characteristic curve (AUROC) as the IUS indices for detecting endoscopic remission (0.82 vs 0.85-0.87) and moderate-to-severe endoscopic activity (0.95 vs 0.94-0.96).^39^ In a similar prospective study of 73 CD patients, IBUS-SAS had the strongest correlation with endoscopic and clinical activity compared with BUSS, SUS-CD, and SUAS-CD.^40^ When comparing AUROC, there was no difference among scores for detecting any endoscopic activity (composite endpoint of SES-CD ≥3 or Rutgeerts ≥i2b), but IBUS-SAS was superior for detecting severe endoscopic activity (composite endpoint of SES-CD ≥9 or Rutgeerts i4). Similarly, a retrospective study of 203 Chinese CD patients observed IBUS-SAS correlated the strongest with endoscopic (SES-CD and Rutgeerts score), biochemical (C-reactive protein), and clinical (Crohn’s Disease Activity Index) parameters compared with BUSS and SUS-CD scores.^41^ With regard to detecting endoscopic activity, all IUS indices had good performance for all IUS scores (AUROC 0.94-0.97), with IBUS-SAS being the highest.^41^

While the accuracy of individual IUS parameters is well established, dedicated studies evaluating the accuracy of IUS indices for specifically detecting POR are limited. In a study of 31 postoperative CD patients, IBUS-SAS, BUSS, and SUS-CD showed moderate correlation with the Rutgeerts score (ρ = 0.55-0.61), with the IBUS-SAS having the strongest correlation (ρ = 0.61).^41^ In another study of 45 postoperative CD patients, Amor Costa et al^42^ demonstrated that the IBUS-SAS, SUS-CD, and SUAS-CD have a high sensitivity in the detection of Rutgeerts ≥i2b. Specifically, the IBUS-SAS was noted to have an AUROC of 87% (sensitivity 90.5%, specificity 70.8% for cutoff ≥28), the SUS-CD an AUROC of 79% (sensitivity 90.5%, specificity 62.5% for cutoff ≥3), and the SUAS-CD an AUROC 80.7% (sensitivity 95.2%, specificity 66.7% for cutoff ≥5.4). Further prospective studies are needed to evaluate whether IUS indices are able to accurately depict severity of POR in comparison with biochemical markers, endoscopy, and other cross-sectional imaging techniques. Additionally, while most data correlate IUS indices to Rutgeerts ≥i2b lesions, clinical studies suggest that Rutgeerts i2a lesions are associated with significantly worse outcomes than i1 or i0, for which IUS data are limited.^43^

## Acute and Chronic Complications Following Ileocolic Resection

Following ICR, 8% to 12% of patients develop intra-abdominal surgical complications (IASCs) within 90 days of surgery.^44^^,^^45^ Common IASCs include anastomotic leak, intra-abdominal phlegmon/abscess, and enterocutaneous fistula. Risk factors for IASCs include nonelective surgery, perioperative smoking, preoperative abdominal sepsis, malnutrition, and preoperative steroid exposure.^46^^,^^47^ IASCs have also been suggested as a risk factor for POR in CD.^45^ Timely identification of IASCs is paramount to prevent progression to life-threatening sepsis and/or anastomosis failure.

**Figure 3.**
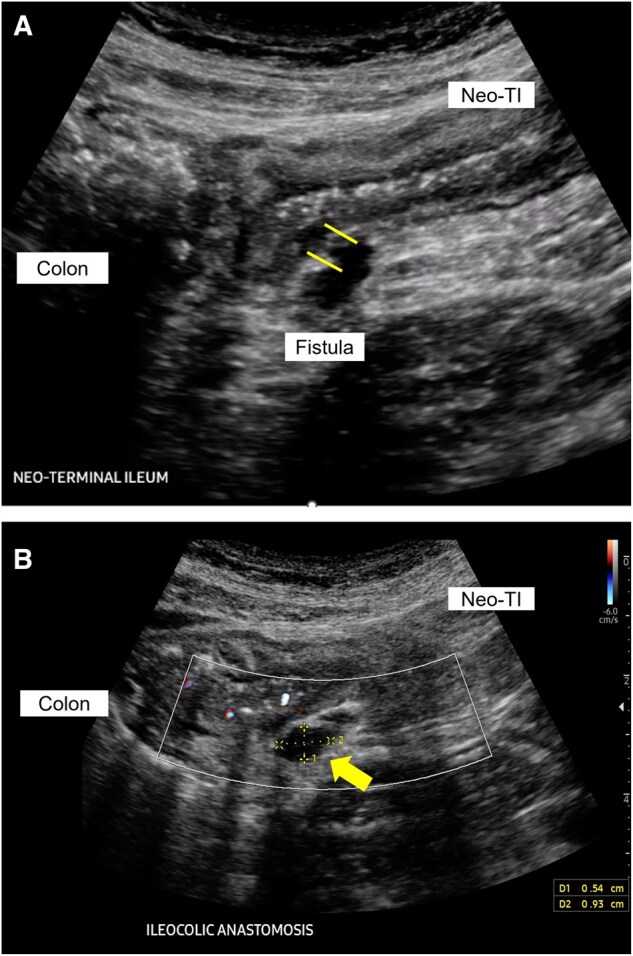
Fistula leading into abscess on IUS. (A) Patient with a fistula (in between lines) arising from the neoterminal ileum (Neo-TI) (B) leading into an intra-abdominal abscess (arrow). There is no color Doppler signal within the dark fluid collection, consistent with an abscess. A cine loop of figure is shown in **Supplementary Media 8.**

While the standard of care for diagnosing anastomotic leaks is with computed tomography utilizing intravenous and oral contrast,^48^ IUS can detect intra-abdominal inflammatory masses and abscesses. On IUS, an abscess or inflammatory mass appears as a hypoechoic fluid mass. An inflammatory mass is differentiated from an abscess by the presence of CDS within the mass.^49^ Intravenous contrast-enhanced ultrasound can aid in differentiating the two but is only routinely performed at few centers by radiologists. In a meta-analysis of 23 studies (n = 3863 subjects), IUS has a pooled sensitivity and specificity of 87% and 97%, respectively, for detecting intra-abdominal inflammatory masses with an accuracy of 91%.^50^  Figure 3 illustrates an example of a patient status post-ICR who developed an intra-abdominal abscess adjacent to the neoterminal ileum. A cine loop (Supplementary Media 8) shows abscess fistulizing from the neoterminal ileum.

**Figure 4.**
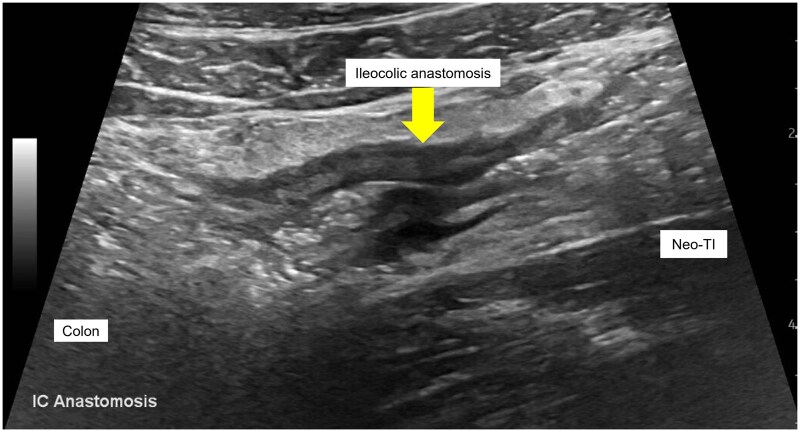
Stenosis of an end-to-side ileocolic anastomosis on IUS. Bowel content is seen traversing a thickened stenosis at the ileocolic anastomosis (arrow) from the neoterminal ileum (right) to the colon (left). IC, ileocolic anastomosis; Neo-TI, neoterminal ileum.

In the context of POR, CD patients can develop CD-related complications such as stenosis of the ICA or neoterminal ileum, fistula, and/or abscess. Because timely intervention for these complications is vital to prevent severe complications, IUS can facilitate prompt detection of these complications. Like surgically naïve CD patients, IUS can accurately detect CD-related complications in postoperative CD. The pooled sensitivity/specificity for detecting CD complications are the following: stenosis, 81%/90%; abscess/inflammatory mass, 87%/95%; and fistula, 67%/97%.^50^ With SICUS, the sensitivity/specificity improves to the following: stenosis, 94%/95%; abscess/phlegmon, 91%/97%; and fistula, 90%/94%. Figure 4 is an example of an ICA stenosis, and Figure 3 is an example of a fistula arising from the neoterminal ileum on IUS.

## POR Monitoring Algorithm

Currently, there is a lack of uniform guidance regarding the optimal timing for the index IUS exam after ICR in CD. Based on expert opinion from an international RAND/UCLA appropriateness study, the index IUS exam can be considered within 3 months after surgery.^51^ For patients in which there is greater concern for early POR, IUS can be performed as early as 4 weeks after surgery. Before 4 weeks, expected postoperative changes such as thickening at the anastomosis are still present, and this can result in challenges with regard to interpretation of findings.

**Figure 5.**
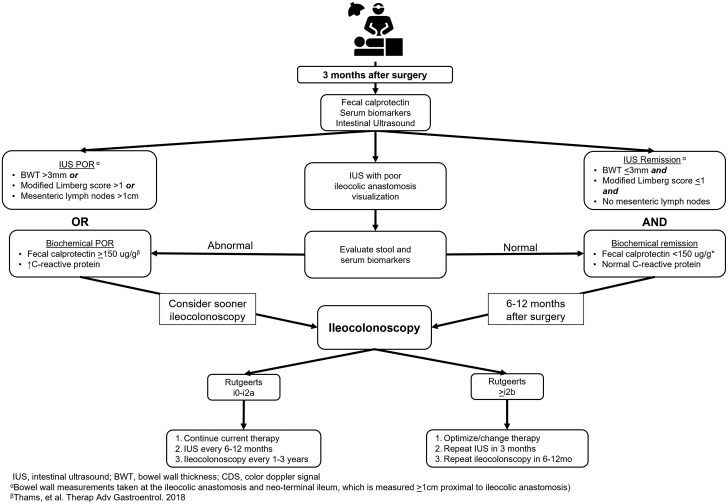
Algorithm for monitoring postoperative Crohn’s disease incorporating intestinal ultrasound (IUS). BWT, bowel wall thickness; POR, postoperative recurrence

As previously noted, a BWT >3 mm is the most significant IUS parameter for detecting postoperative POR of CD. When combined with FCP, the specificity increases to 93%^14^ Hence, pairing IUS with FCP at 3 months postoperatively with routine bloodwork, including C-reactive protein, optimizes the accuracy for detecting POR. FCP at 3 months after surgery in CD has been shown to be significantly higher in those with POR compared with those without.^52^ While the optimal FCP cutoff for POR has yet to be investigated in prospective trials, FCP ≥150µg/g is found to have the best overall accuracy.^53^ On the index postoperative evaluation, if there is any abnormality detected based on IUS, FCP, and/or labs (ie, decreased hemoglobin, elevated C-reactive protein), we suggest that the clinician should consider referral for a sooner ileocolonoscopy than the recommended 6 to 12 months after surgery.^7^ This serves 2 purposes: (1) detecting early POR that allows for timely intervention and (2) pairing baseline IUS and endoscopic findings to inform future evaluations. If IUS and FCP are normal, the first postoperative ileocolonoscopy should still be performed within 6 to 12 months after surgery. If significant POR ­(Rutgeerts ≥i2b) is not detected on the index postoperative colonoscopy, the ICA and neoterminal ileum can be monitored with IUS and FCP every 6 months and adjusted according to subsequent results. Ileocolonoscopy every at least 1 to 3 years is recommended for definitive POR monitoring.^54^ If significant POR is detected on IUS, then the standard IUS monitoring algorithm for escalating or starting new therapy should be followed.^55^  Figure 5 outlines a recommended postoperative monitoring algorithm for patients with CD.

## Limitations of IUS for Monitoring Postoperative CD and Future Directions

Despite its excellent utility in the postoperative monitoring of CD, IUS has inherent limitations that warrant consideration. First, the accuracy of IUS to detect POR can be impacted by operator experience, location of the anastomosis, and abdominal/body habitus. Additionally, acquisition protocols are currently not standardized, which may impact the images obtained. Furthermore, as different IUS scoring systems require inclusion of differing number of IUS parameters, use of highly predictive scores such as IBUS-SAS requires increased level of experience and thus is the most operator dependent. As such, use of simpler scores with acceptable sensitivity and specificity such as SES-CD or SUS-CD in combination with another modality such as serum and stool biomarkers of inflammation may prove paramount for accurately detecting and monitoring for POR in CD. Additionally, it should be noted that several cutoff values for IUS scores have been utilized in the literature to define active disease, with a definite consensus that balances sensitivity and specificity yet to be determined. Segmental limitations to IUS should also be noted, with some studies reporting SUS-CD and IBUS-SAS to be less accurate in predicting endoscopic activity at the terminal ileum compared with colon.^56^ In terms of the postoperative setting, no standardized scoring system or established IUS parameter cutoffs that exist for evaluating the ICA itself. In terms of the ability to detect clinically meaningful changes over time, limited data exist for IUS scores. While BUSS has demonstrated sensitivity to change in response to therapy and correlates with endoscopic improvement over time,^36^ evidence for other IUS scores such as SUS-CD and IBUS-SAS is sparse.

It should be noted that current IUS disease activity indices for CD are derived from surgically naïve subjects. As Rutgeerts i2a and i2b are associated with different prognostic significance, further studies are needed to determine the clinical significance of increased BWT and/or presence of CDS at the anastomosis without abnormalities in the proximal neoterminal ileum. This also highlights the lack of guidance and consensus as to the location of measurements on diagnostic imaging. Establishing these parameters is important to avoid under- or overtreatment of CD based on IUS findings. Additionally, while IUS having excellent interrater reliability for nonsurgical CD is well established, the interrelated reliability of evaluating the ICA and neoterminal ileum on IUS has not been rigorously evaluated to the best of our knowledge. This gap in knowledge is important to address to better understand the strengths and limitation of IUS in the algorithm of monitoring postoperative CD. Also, IUS can reliably identify the ileocolic anastomosis in most cases, but detecting and monitoring recurrence at an enteroenteric anastomosis can be challenging if it is in the proximal bowel, where IUS has lower sensitivity for detecting active CD compared with the terminal ileum and colon.^57^ For these patients, assessment of the anastomosis with CTE or MRE, capsule endoscopy, and/or double balloon enteroscopy are important adjuncts. Finally, the recommendation to perform IUS 3 months postoperatively is based on expert consensus, and there is no evidence to support the optimal timing of the first postoperative IUS. The ongoing INSIGHT study (NCT05713409) aims to address this gap in knowledge and determine if early IUS and FCP can predict endoscopic POR.

Several gaps in knowledge remain regarding IUS for monitoring POR. First, the optimal timing of the first postoperative IUS and subsequent IUS exam for detecting and monitoring POR is poorly understood. The INSIGHT study will offer valuable insight into this gap, but clinical impact of very early detection of POR also needs to be investigated. Second, studies are needed to establish early IUS predictors of POR because some alterations like mild vascularization at the anastomosis may be normal during the first couple of months after surgery. Finally, validated IUS scoring indices specific for POR need to be developed to support accurate and reliable detection of POR and its severity. Although IUS is already a valuable tool for detecting and monitoring POR, addressing these gaps in knowledge will be important for understanding how to optimally integrate IUS in the management of postoperative CD.

## Conclusions

In summary, IUS is an accurate and noninvasive tool for monitoring postoperative CD. IUS is well tolerated and cost-effective and offers real-time bedside evaluation to facilitate prompt clinical decision making to address POR and prevent postoperative complications. While the technical performance of IUS in postoperative CD patients is comparable to IUS performed in native anatomy, utilization of the low-frequency transducer is recommended to accurately map the postsurgical anatomy, and high-frequency transducers can be utilized to capture more detailed measurements. Although further studies are required to establish definitive cutoff values for detecting clinically significant POR, a BWT >3 mm and/or FCP >150 µg/g^53^ should prompt consideration of early ileocolonoscopy to assess for POR and determine its severity.

## Supplementary Material

izaf248_Supplementary_Data
