# Supplementary material for: Intestinal Ultrasound for Monitoring Postoperative Crohn’s Disease: A Review and Visual Atlas
Source: Inflamm Bowel Dis. 2025 Dec 3;32(4):755–64. doi: 10.1093/ibd/izaf248 (PMC13046064; doi:10.1093/ibd/izaf248)
Supplement: izaf248_Supplementary_Data [file izaf248_supplementary_data.zip › Supplementary files.docx]

**Supplementary Figure 1**. Initial localization of the ileocolic anastomosis with curvilinear probe.
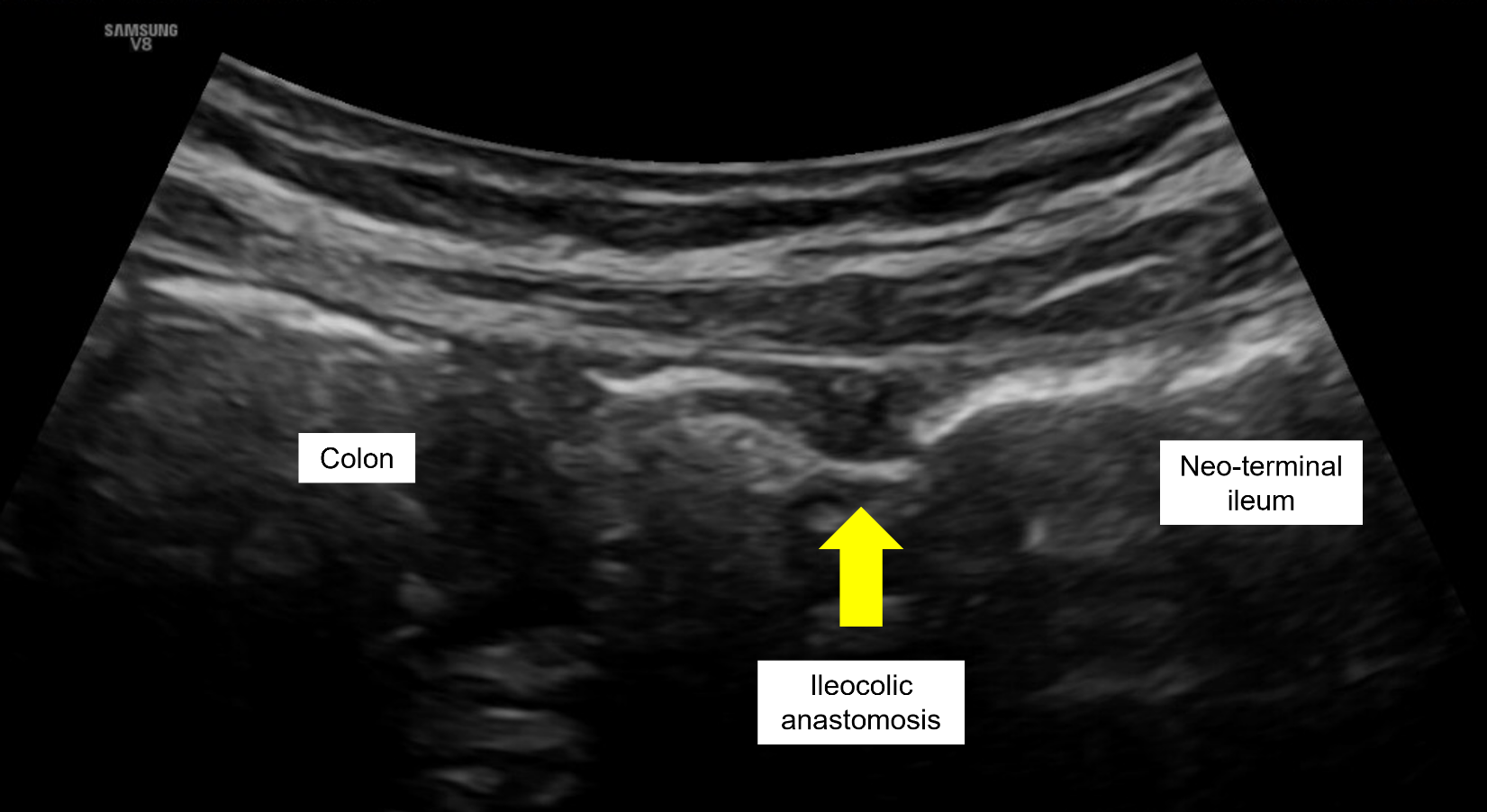


With the curvilinear probe, bowel contents (arrow) is visualized entering the distal colon (left) from the neo-terminal (right).

**Supplementary Media 1-2**. Cine clips of side-to-side anastomoses.

**Supplementary Media 3-4**. Cine clips of end-to-side anastomoses.

**Supplementary Media 5**. Cine clip of small intestine contracted ultrasound (SICUS).

**Supplementary Media 6.** Cine clip of enteroenteric anastomosis.

**Supplementary Media 7.** Cine clip of colocolonic anastomosis.

**Supplementary Media 8.** Cine slip of intra-abdominal abscess fistulizing from the neo-terminal ileum.

**Supplementary Figure 2.** Still images of enteroenteric anastomosis.
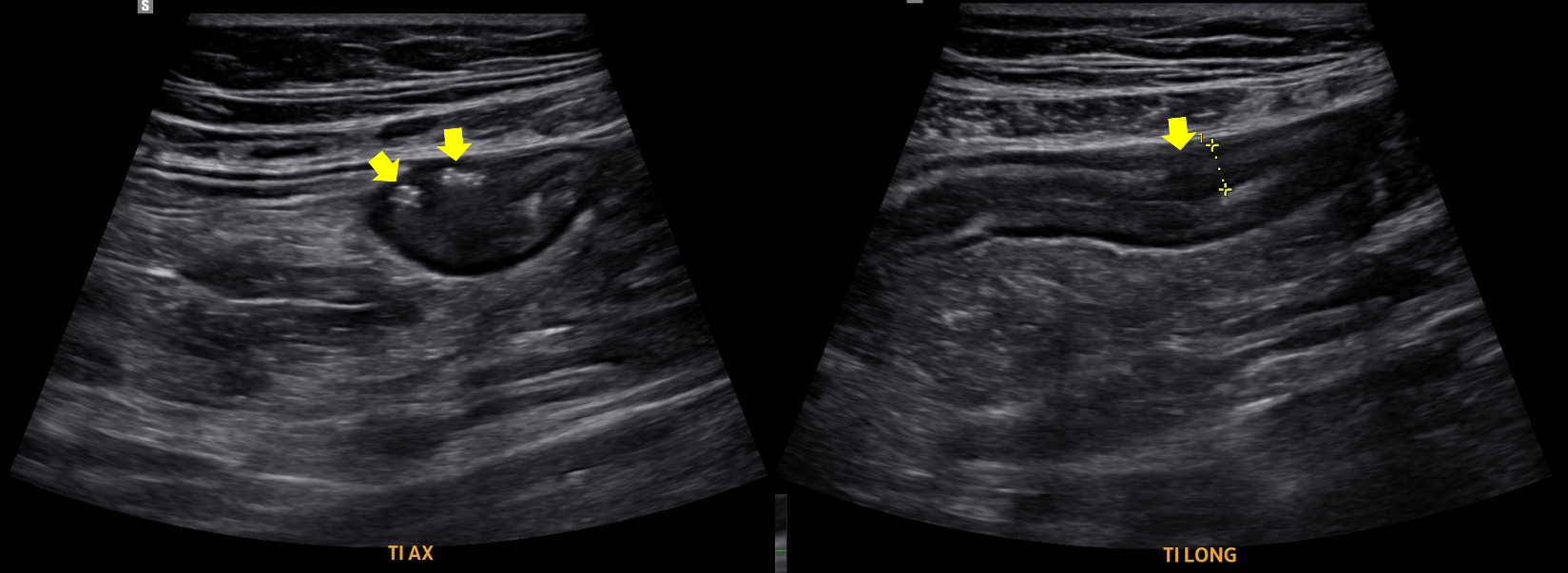


In the left panel, the yellow arrow points to the staple line of the anastomosis. In the right panel, the yellow arrow points to the short defect within the bowel wall associated with the enteroenteric anastomosis.

**Supplementary Table 1**. Segmental intestinal ultrasound scores for Crohn’s disease.

| **IUS Score** | **Components** |
| --- | --- |
| SUS-CD^36^ | BWT + CDS   - BWT scored as 0 (≤3.0mm), 1 (3.0-4.9 mm), 2 (5.0-7.9 mm), or 3 (≥8.0mm)   CDS scored as 0 (no or single vessel), 1 (2-5 vessels/cm^2^), or 2 (>5 vessels/cm^2^) |
| SUAS-CD^37^ | BWT + CDS   - BWT scored in mm - CDS scored as 0 (absent), 1 (1–2 vessels/cm^2^), 2 (3–5 vessels/cm^2^), or 3 (>5 vessels per cm^2^) |
| BUSS^38^ | 0.75*BWT + 1.65*CDS   - BWT scored in mm - CDS scored as 0 (absent) or 1 (present) – the worst bowel segment to be used for scoring |
| SPAUSS^39^ | BWT + iFat + CDS   - BTW scored as 1 (<3.9mm), 4 (4.0-6.9mm), or 6 (>7mm) - i-fat scored as 0 (absent), 1 (mild), or 6 (moderate/severe) - CDS scored as 0 (absent), 1 (mild), or 2 (moderate/severe) |
| IBUS-SAS^40^ | 4*BWT + 15*iFat + 7*CDS + 4*BWS   - BWT scored in mm - i-fat scored as 0 (absent), 1 (uncertain), or 2 (present) - BWS scored as 0 (normal), 1 (uncertain), 2 (focal disruption ≤3 cm), or 3 (extensive disruption >3cm),   CDS scored as 0 (absent), 1 (short signals), 2 (long signals inside the wall), or 3 (long signal inside and outside the wall) |
| BWT, bowel wall thickness; CDS, color doppler signal; iFat, inflammatory mesenteric fat; BWS, bowel wall stratification | |
